# *Anaplasma phagocytophilum*, *Bartonella* spp*.*, haemoplasma species and *Hepatozoon* spp*.* in ticks infesting cats: a large-scale survey

**DOI:** 10.1186/s13071-018-2789-5

**Published:** 2018-03-20

**Authors:** Florent Duplan, Saran Davies, Serina Filler, Swaid Abdullah, Sophie Keyte, Hannah Newbury, Chris R. Helps, Richard Wall, Séverine Tasker

**Affiliations:** 1 Small Animal Hospital, Langford Vets, University of Bristol, Langford, United Kingdom; 2 Veterinary Parasitology and Ecology Group, School of Biological Sciences, University of Bristol, Bristol, United Kingdom; 3 Bristol Veterinary School, University of Bristol, Langford, United Kingdom; 4 MSD Animal Health, Walton Manor, Walton, Milton Keynes, United Kingdom; 5 Molecular Diagnostic Unit, Diagnostic Laboratories, Langford Vets, University of Bristol, Langford, United Kingdom

**Keywords:** Feline, Tick-borne pathogens, *Anaplasma phagocytophilum*, *Bartonella henselae*, *Bartonella clarridgeiae*, Haemoplasma, *Mycoplasma haemofelis*, “*Candidatus* Mycoplasma haemominutum”, “*Candidatus* Mycoplasma turicensis”, *Hepatozoon felis*, *Hepatozoon silvestris*

## Abstract

**Background:**

Ticks derived from cats have rarely been evaluated for the presence of pathogens. The aim of this study was to determine the prevalence of *Anaplasma phagocytophilum*, *Bartonella* spp*.*, haemoplasma species and *Hepatozoon* spp. in ticks collected from cats in the UK.

**Methods:**

Five hundred and forty DNA samples extracted from 540 ticks collected from cats presenting to veterinarians in UK practices were used. Samples underwent a conventional generic PCR assay for detection of *Hepatozoon* spp. and real-time quantitative PCR assays for detection of *Anaplasma phagocytophilum* and three feline haemoplasma species and a generic qPCR for detection of *Bartonella* spp. Feline *28S* rDNA served as an endogenous internal PCR control and was assessed within the haemoplasma qPCR assays. Samples positive on the conventional and quantitative generic PCRs were submitted for DNA sequencing for species identification.

**Results:**

Feline *28S* rDNA was amplified from 475 of the 540 (88.0%) ticks. No evidence of PCR inhibition was found using an internal amplification control. Of 540 ticks, 19 (3.5%) contained DNA from one of the tick-borne pathogens evaluated. Pathogens detected were: *A. phagocytophilum* (*n* = 5; 0.9%), *Bartonella* spp. (*n* = 7; 1.3%) [including *Bartonella henselae* (*n* = 3; 0.6%) and *Bartonella clarridgeiae* (*n* = 1; 0.2%)], haemoplasma species (*n* = 5; 0.9%), “*Candidatus* Mycoplasma haemominutum” (*n* = 3; 0.6%), *Mycoplasma haemofelis* (*n* = 1; 0.2%), “*Candidatus* Mycoplasma turicensis” (*n* = 1; 0.2%), *Hepatozoon* spp. (*n* = 2; 0.4%), *Hepatozoon felis* (*n* = 1; 0.2%) and *Hepatozoon silvestris* (*n* = 1; 0.2%).

**Conclusion:**

These data provide important information on the prevalence of tick-borne pathogens in ticks infesting cats, with the identification of haemoplasma species, *A. phagocytophilum*, *H. felis* and *Bartonella* spp. (including *B. henselae* and *B. clarridgeiae*). This study also documents the first report of *H. silvestris* in ticks collected from domestic cats.

## Background

Ticks are important arthropod vectors that transmit a wide range of viral, bacterial and protozoan pathogens [1]. Tick-borne pathogens are transmitted to the host mostly by tick bites, although tick ingestion is also a possible route of transmission [1]. Their prolonged periods of feeding and large blood meals allow important numbers of pathogens to be transmitted and this, together with their high rates of reproduction and possible pathogen transmission between tick life-cycle stages (trans-stadial) and generations (trans-ovarial) [2], make ticks efficient vectors.

A recent study revealed that the most common tick species found on cats in the UK were (in decreasing order of prevalence) *Ixodes ricinus*, *Ixodes hexagonus* and *Ixodes trianguliceps*, with an overall prevalence of tick attachment on cats of 6.6% [3]. A similar study of ticks found on dogs in the UK revealed the presence of *I. ricinus* and *I. hexagonus*, but *Ixodes canisuga, Haemaphysalis punctata* and *Dermacentor reticulatus* were also reported, together with a much higher prevalence (30%) of tick infestation on dogs [4]. In line with the lower prevalence of tick infestation in cats compared to dogs, it is also thought that transmission of tick-borne pathogens is likely to be less common in cats than in dogs, although there is a lack of publications in this field [5]. Possible explanations for the species discrepancies are: differences in lifestyle, behaviour (e.g. increased self-grooming in cats compared to dogs) and in immunity to tick-borne infections [5]. Nevertheless, tick-borne pathogens are reported in cats and can be problematic; the pathogen species reported include *Babesia* spp., *Hepatozoon* spp., *Borrelia* spp., *Ehrlichia* spp., *Anaplasma* spp., haemoplasma species and *Bartonella* spp. [6].

The aim of this study was to determine the prevalence of selected tick-borne pathogens in ticks collected from cats in a large-scale national surveillance study. The tick-DNA samples analysed in the present study had previously been assessed for the presence of *Borrelia* spp. and *Babesia* spp. DNA [3]. The pathogens evaluated were *A. phagocytophilum*, *Bartonella* spp.*, Hepatozoon* spp. and three haemoplasma species.

## Methods

### Tick samples

DNA samples obtained from 540 (308 *I. ricinus*, 224 *I. hexagonus* and 8 *I. trianguliceps*) ticks that were collected from 540 cats between May and October 2016 by veterinary surgeons throughout the UK as part of a national surveillance study, details of which have been published previously [3], were used in the current study. As previously described [3], these ticks had been collected by veterinarians from 278 veterinary practices and most ticks (440) were adults and most (535) were female; 122 were fully fed, 372 partially fed and 46 unfed. An internal amplification control (IAC) had been spiked into the tick samples before DNA extraction to monitor for successful extraction and the absence of PCR inhibitors by subsequent quantitative (q) PCR analysis of the IAC, as previously described [3].

### *Anaplasma phagocytophilum* real-time qPCR

*Anaplasma phagocytophilum* DNA was detected using a qPCR for the *msp2* gene [7] modified as follows: each qPCR consisted of GoTaq Hot Start Mastermix (Promega, Southampton, UK), MgCl_2_ to a final concentration of 4.5 mM, forward and reverse primers and TaqMan probe (Table 1) at a final concentration of 100 nM each, 2 μl of template DNA, and water to a final volume of 10 μl. Thermal cycling conditions comprised 95 °C for 2 min and 45 cycles of 95 °C for 15 s, and 60 °C for 30 s (Agilent MX3005P qPCR, Agilent, Stockport, UK). Fluorescence data were collected at 516 nm at the end of each annealing/extension step. A positive control sample (of known copy number) and negative control (water) were included on each plate.

**Table 1:** Details of the qPCR/PCR assays used in the study for the detection of tick-borne pathogens

Target species (target gene)	PCR primer and probe sequences (5'–3')	Product size (bp)	Reference
*Anaplasma phagocytophilum* (*msp2*)	F: ATGGAAGGTAGTGTTGGTTATGGTATT	77	[7]
	R: TTGGTCTTGAAGCGCTCGTA		
	FAM-TGGTGCCAGGGTTGAGCTTGAGATTG-BHQ1		
*Bartonella henselae* (*alr-gcvP* intergenic spacer)	F: GAGGGAAATGACTCTCTCAGTAAAA	110	[9]^a^
	R: TGAACAGGATGTGGAAGAAGG		
	FAM-CAGCCAAATATACGGGCTATCCATCAA-BHQ1		
*Bartonella* spp. (*ssrA*)	F: GCTATGGTAATAAATGGACAATGAAATAA	299	[8]^b^
	R: GGCTTCTGTTGCCAGGTG		
	FAM-ACCCCGCTTAAACCTGCGACG-BHQ1		
“*Candidatus* Mycoplasma haemominutum” (*16S* rRNA gene)	F: TGATCTATTGTKAAAGGCACTTGCT	135	[10]
	R: TTAGCCTCYGGTGTTCCTCAA		
	FAM-TTCAATGTGTAGCGGTGGAATGCGT-BHQ1		
“*Candidatus* Mycoplasma turicensis” (*16S* rRNA gene)	F: AGAGGCGAAGGCGAAAACT	138	[10]
	R: ACGTAAGCTACAACGCCGAAA		
	FAM-CGTAAACGATGGGTATTAGATGTCGGGAT-BHQ1		
Feline genomic DNA (*28S* rRNA)	F: AGCAGGAGGTGTTGGAAGAG	100	[10]
	R: AGGGAGAGCCTAAATCAAAGG		
	Texas Red-TGG CTT GTG GCA GCC AAG TGT-BHQ2		
*Hepatozoon*. spp. (*18S* rRNA gene)	F: AAACGGCTACCACATNTAAGGA	522	[11]
	R: AATACAAATGCCCCCAACTNT		
*Mycoplasma haemofelis* (*16S* rRNA gene)	F: GTGCTACAATGGCGAACACA	80	[10]
	R: TCCTATCCGAACTGAGACGAA		
	FAM-TGTGTTGCAAACCAGCGATGGT-BHQ1		

^a^The reverse and probe sequences in the original paper are incorrectly labelled; the correct sequences are cited in this table

^b^The reverse primer has been modified compared to the one described in the paper

*Abbreviations*: *F*, forward primer sequence; *R*, reverse primer sequence; *FAM*, 6-carboxyfluorescein; *BHQ*, black hole quencher (1 or 2 as indicated)

### *Bartonella* spp. qPCRs and sequencing

*Bartonella* spp. were detected using a qPCR targeting a fragment of the *ssrA* gene [8] modified as follows: each qPCR reaction consisted of GoTaq Hot Start Mastermix, MgCl_2_ to a final concentration of 4.5 mM, forward and reverse primers at a final concentration of 500 nM each and TaqMan probe at a final concentration of 100 nM (Table 1), 2 μl of template DNA and water to a final volume of 10 μl. The thermal cycling protocol consisted of an initial denaturation at 95 °C for 2 min and 40 cycles of 95 °C for 15 s and 60 °C for 30 s (Agilent MX3005P qPCR, Agilent, Stockport, UK). Fluorescence data were collected at 516 nm at the end of each annealing/extension step. A positive control sample (of known copy number) and negative control (water) were included on each plate.

All samples positive on the *Bartonella* spp. qPCR were then screened using a *B. henselae* specific qPCR targeting the alr-gcvP intergenic spacer [9] modified as follows: each qPCR reaction consisted of GoTaq Hot Start Mastermix, MgCl_2_ to a final concentration of 4.5 mM, forward and reverse primers and TaqMan probe (Table 1) at a final concentration of 100 nM each, 2 μl of template DNA and water to a final volume of 10 μl. The thermal cycling conditions and controls used were identical to those described above for the *Bartonella* spp. qPCR.

All samples positive for *Bartonella* spp. but negative for *B. henselae* underwent repeat amplification using the *Bartonella* spp. qPCR assay (as above) in a final volume of 25 μl. The PCR amplicons were prepared for DNA sequencing using a Nucleospin® 96 PCR Clean-up Core Kit (Macherey-Nagel, Düren, Germany) and submitted to a commercial sequencing laboratory (DNA Sequencing & Services, MRC I PPU, School of Life Sciences, University of Dundee, UK) using Applied Biosystems Big-Dye Ver 3.1 chemistry on an Applied Biosystems model 3730 automated capillary DNA sequencer.

### Haemoplasma species qPCRs

Feline haemoplasma DNA was detected using individual species-specific qPCRs targeting the *16S* rRNA gene each of *M. haemofelis*, “*Ca.* M. haemominutum” and “*Ca.* M. turicensis”, as previously described [10]. Each haemoplasma species qPCR was also duplexed with a qPCR for the detection of feline *28S* rDNA as an internal control for feline blood (Table 1), again as previously described [10]. The qPCR assay for each species consisted of GoTaq Hot Start Mastermix, MgCl_2_ to a final concentration of 4.5 mM, forward and reverse primers (for each species as shown in Table 1) at a final concentration of 200 nM each, TaqMan probe (for each species as shown in Table 1) at 50 nM, 2 μl of template DNA and water to a final volume of 10 μl. A positive control sample (of known copy number) and negative control (water) were included on each plate. The thermal cycling conditions were identical to those described above for the *Bartonella* spp. qPCR.

### *Hepatozoon* spp*.* PCR and sequencing

*Hepatozoon* spp. DNA was detected using a conventional PCR targeting the *18S* rRNA gene as previously described [11]. Each PCR consisted of GoTaq Hot Start Mastermix, forward and reverse primers (Table 1) at a final concentration of 200 nM each, 2 μl of template DNA and water to a final volume of 10 μl. A positive control sample and negative control (water) were included on each plate. The thermal cycling protocol consisted of an initial denaturation at 95 °C for 2 min, then 40 cycles of 95 °C for 15 s and 60 °C for 45 s (BioRad DNA Engine PTC-200, BioRad, Watford, UK). Agarose gel electrophoresis was used to visualize target amplicons. Positive samples were identified as a defined band of approximately 522 bp on the gel.

Samples positive for *Hepatozoon* spp. underwent repeat amplification using the *Hepatozoon* spp. assay (as above) in a final volume of 25 μl, amplicons were purified and submitted for DNA sequencing, as described above.

### Data handling

Data was entered into Microsoft Excel (version 15.32) and descriptive statistics obtained. Coordinates were generated by converting owner postcodes. The WGS84 (World Geodetic System) was used to map the location of each sample in QGIS (version 2.18.2). Sequence data were edited and analysed in BioEdit Sequence Alignment Editor (version 7.2.5). Output sequences were BLAST searched against the NCBI GenBank sequence database to determine the species present (www.ncbi.nlm.nih.gov/BLAST/).

## Results

### Analysis of the controls

The IAC was successfully amplified in all samples following qPCR as previously described [3]. Feline *28S* rDNA was amplified from 475 of the 540 (88%) tick samples. Details of the feline *28S* rDNA results according to tick feeding status (i.e. fully fed, partially fed and unfed) are shown in Table 2.

**Table 2:** Feline *28S* rDNA PCR results according to tick feeding status

Tick feeding status	Positive PCR result (%)	Negative PCR result (%)	Total
Fully fed	102 (83.6)	20 (16.4)	122
Partially fed	340 (91.4)	32 (8.6)	372
Unfed	33 (71.7)	13 (28.3)	46

### Prevalence and geographical location of the different pathogens

**Fig 1:**
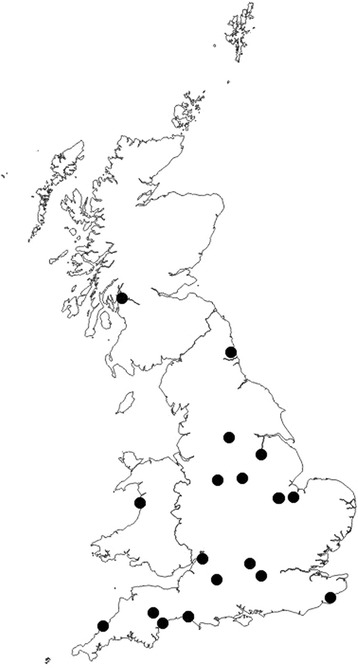
Location of all ticks positive by PCR for any of the selected tick-borne pathogens

Of the 540 DNA samples from ticks, 19 (3.5%) were positive by PCR/qPCR for DNA of one of the pathogens described; no tick was positive for DNA from more than one pathogen. The pathogens detected were widely distributed throughout the UK (Fig. 1).

### *Anaplasma phagocytophilum*

**Fig 2:**
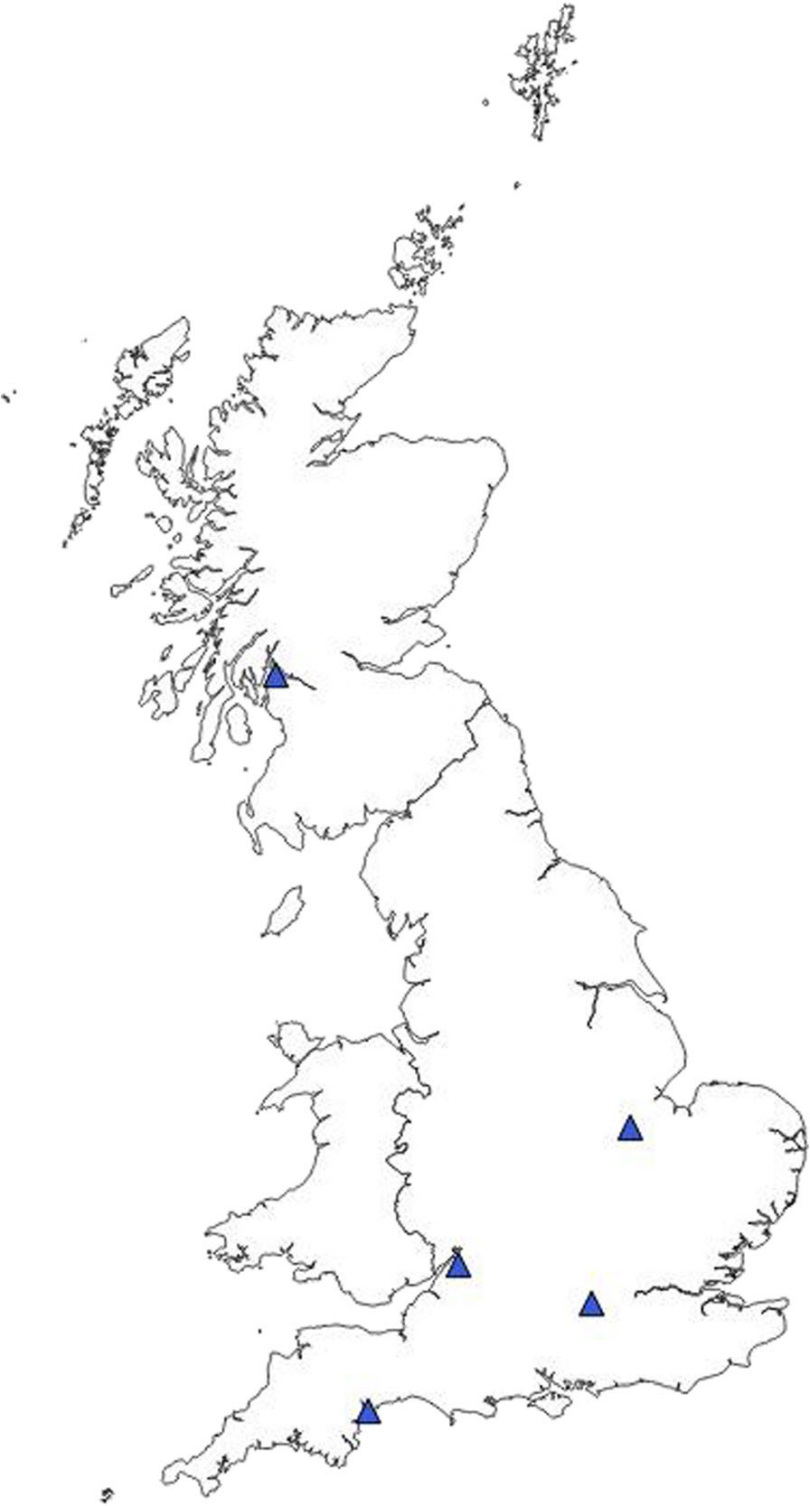
Location of ticks positive by qPCR for *A. phagocytophilum* DNA.

Five of the 540 ticks (0.9%) were positive by qPCR for *A. phagocytophilum* DNA*.* Ticks positive for *A. phagocytophilum* DNA were widely spread throughout the UK (Fig. 2). The positive ticks comprised four *I. ricinus* and one *I. hexagonus* (Table 3).

**Table 3:** Prevalence, tick species identified, method of detection, sequence identity and sequence identity information

Pathogen		Prevalence (%) (*n* = 540)	Tick species (*n*)	Method of detection	Sequence identity (%)	GenBank ID
*A. phagocytophilum*		5 (0.9)	*I. hexagonus* (1)	qPCR	na	na
			*I. ricinus* (4)			
*Bartonella* spp.	*B. henselae*	3 (0.6)	*I. hexagonus* (2)	qPCR	na	na
			*I. ricinus* (1)			
	*B. clarridgeiae*	1 (0.2)	*I. hexagonus* (1)	qPCR	96 (based on 225 bp)	HG519012.1
	Other species	3 (0.6)	*I. hexagonus* (3)		na	na
Haemoplasma species	“*Ca.* M. haemominutum”	3 (0.6)	*I. trianguliceps* (1)	qPCR	na	na
			*I. ricinus* (2)			
	*M. haemofelis*	1 (0.2)	*I. trianguliceps* (1)			
	“*Ca.* M. turicensis”	1 (0.2)	*I. ricinus* (1)			
*Hepatozoon* spp*.*	*H. felis*	1 (0.2)	*I. hexagonus* (1)	PCR	90 (based on 315 bp)	KY215817.1
	*H. silvestris*	1 (0.2)	*I. ricinus* (1)		100 (based on 452 bp)	KX757032.1

*Abbreviations*: *na*, not applicable; *PCR*, conventional polymerase chain reaction; *qPCR*, quantitative polymerase chain reaction

### *Bartonella* spp.

**Fig 3:**
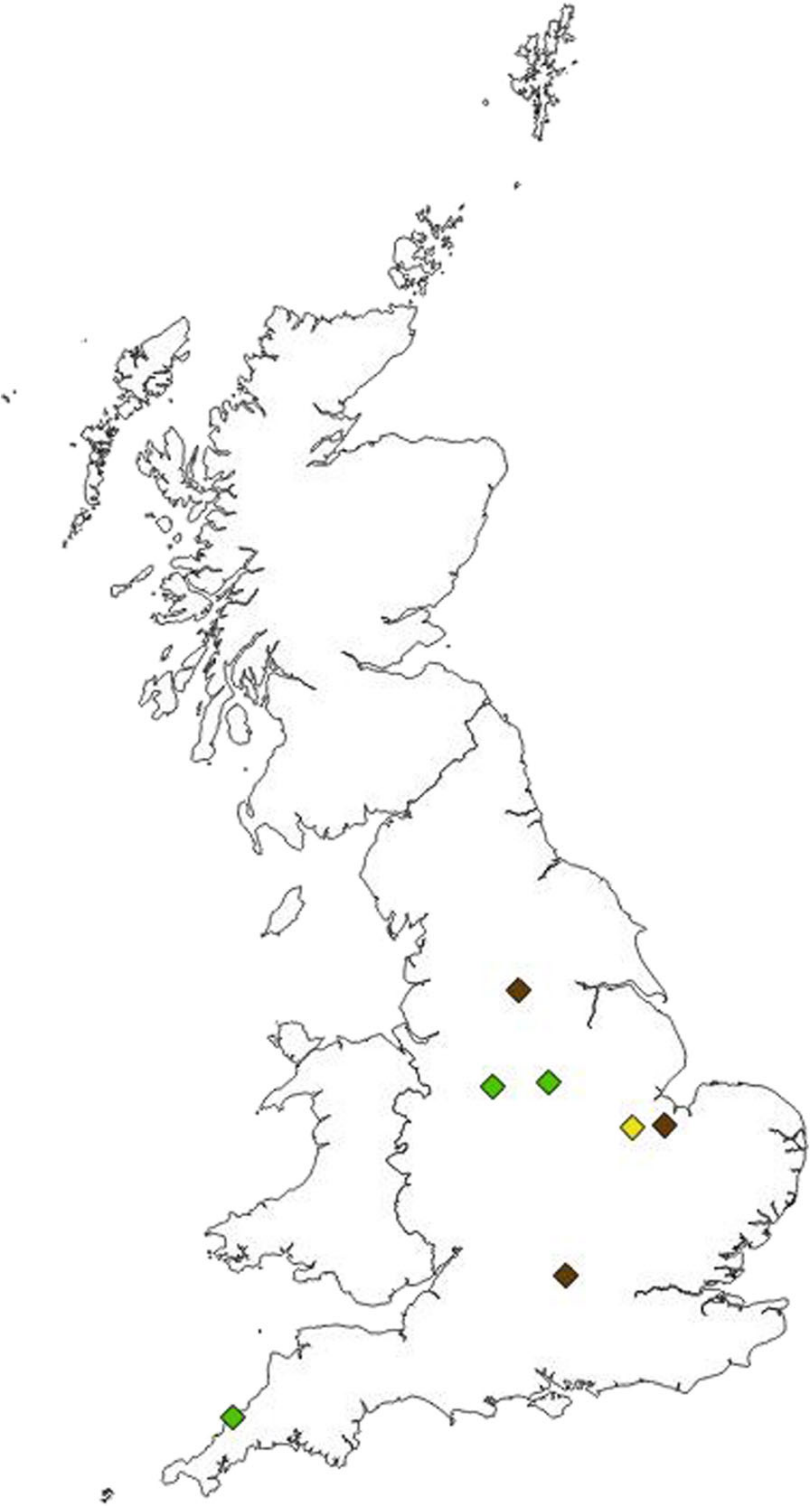
Location of ticks positive by qPCR for *Bartonella* species DNA. *Key*: Green diamond, *B. henselae*; yellow diamond, *B. clarridgeiae*; brown diamond, *Bartonella* spp. (unable to identify to the species level)

Seven of the 540 ticks (1.3%) were positive by qPCR for *Bartonella* spp*.* DNA (Table 1)*.* Ticks positive for *Bartonella* spp*.* DNA were widely spread throughout the UK (Fig. 3). Three (0.6% of the total population of 540 ticks) of the seven ticks, two *I. hexagonus* and one *I. ricinus*, were positive by qPCR for *B. henselae* DNA (Table 3). One of remaining four ticks (0.2% of total population), an *I. hexagonus*, was found to be positive for *B. clarridgeiae* DNA following sequencing, with 96% sequence identity to a previously published sequence in GenBank (*B. clarridgeiae*, HG519012) (Table 3). Sequencing failed for the other three tick samples positive on the *Bartonella* spp*.* qPCR but negative for the *B. henselae* qPCR, despite the repeat *Bartonella* spp*.* qPCR still yielding a positive result, and thus no species identification could be made in these three samples.

### Haemoplasma species

**Fig 4:**
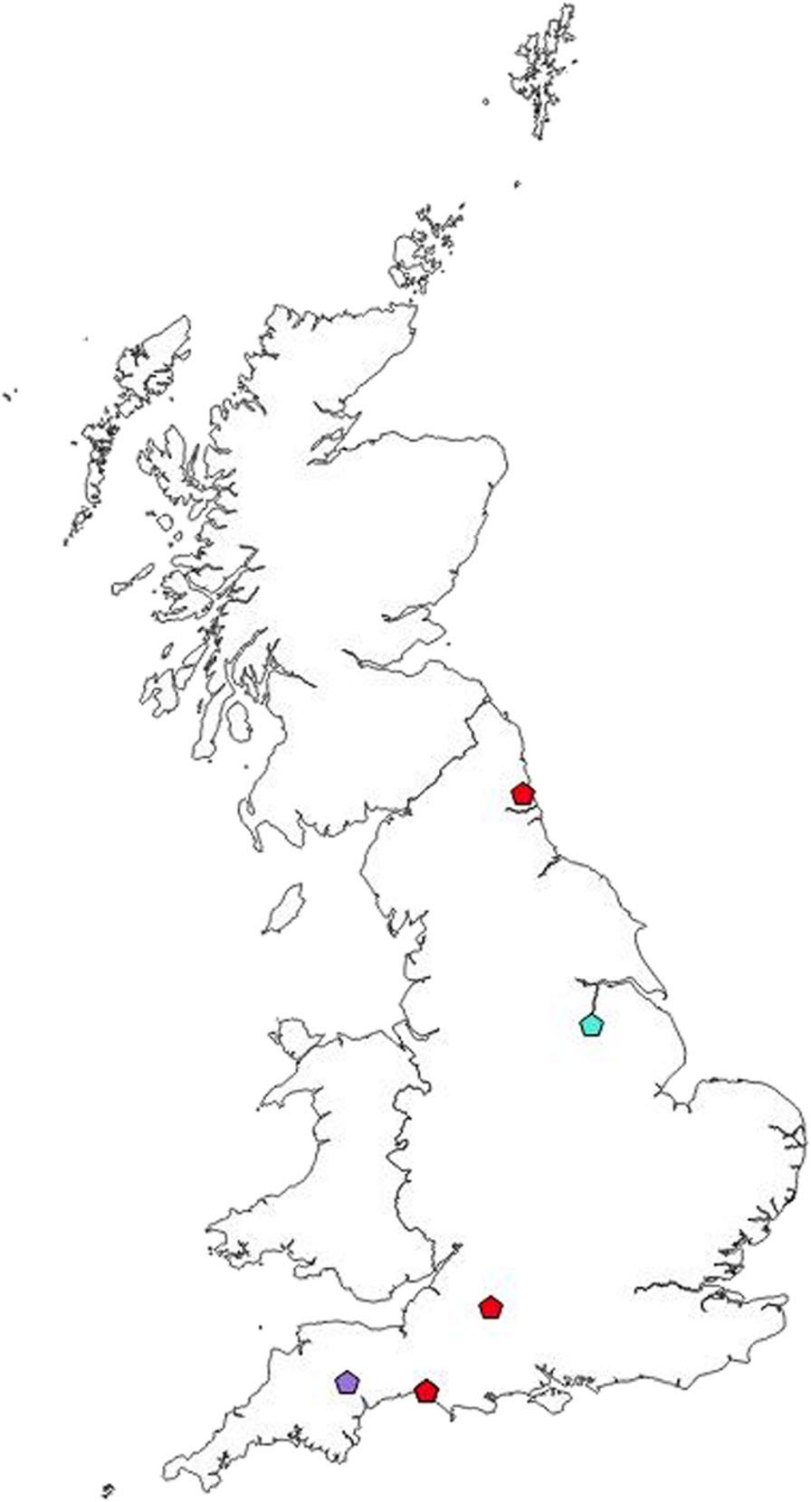
Location of ticks positive by qPCR for feline haemoplasmas. *Key*: Red pentagon, “*Ca.* M. haemominutum”; turquoise pentagon: *M. haemofelis*, purple pentagon: “*Ca.* M. turicensis”

Five of the 540 ticks (0.9%) were positive by qPCR for haemoplasma DNA (Table 1)*.* Ticks positive for haemoplasma DNA were widely spread throughout the UK (Fig. 4). Three ticks (0.6%, 3/540), two *I. ricinus* and one *I. trianguliceps*, were positive by qPCR for “*Ca.* M. haemominutum” (Table 3). One *I. trianguliceps* (0.2%) was positive by qPCR for *M. haemofelis* (Table 3)*.* One *I. ricinus* (0.2%) was positive by qPCR for “*Ca.* M. turicensis” (Table 3). Interestingly, of the *I. trianguliceps* ticks in this study, 25% (2/8) were positive by qPCR for haemoplasma DNA.

### *Hepatozoon* spp.

**Fig 5:**
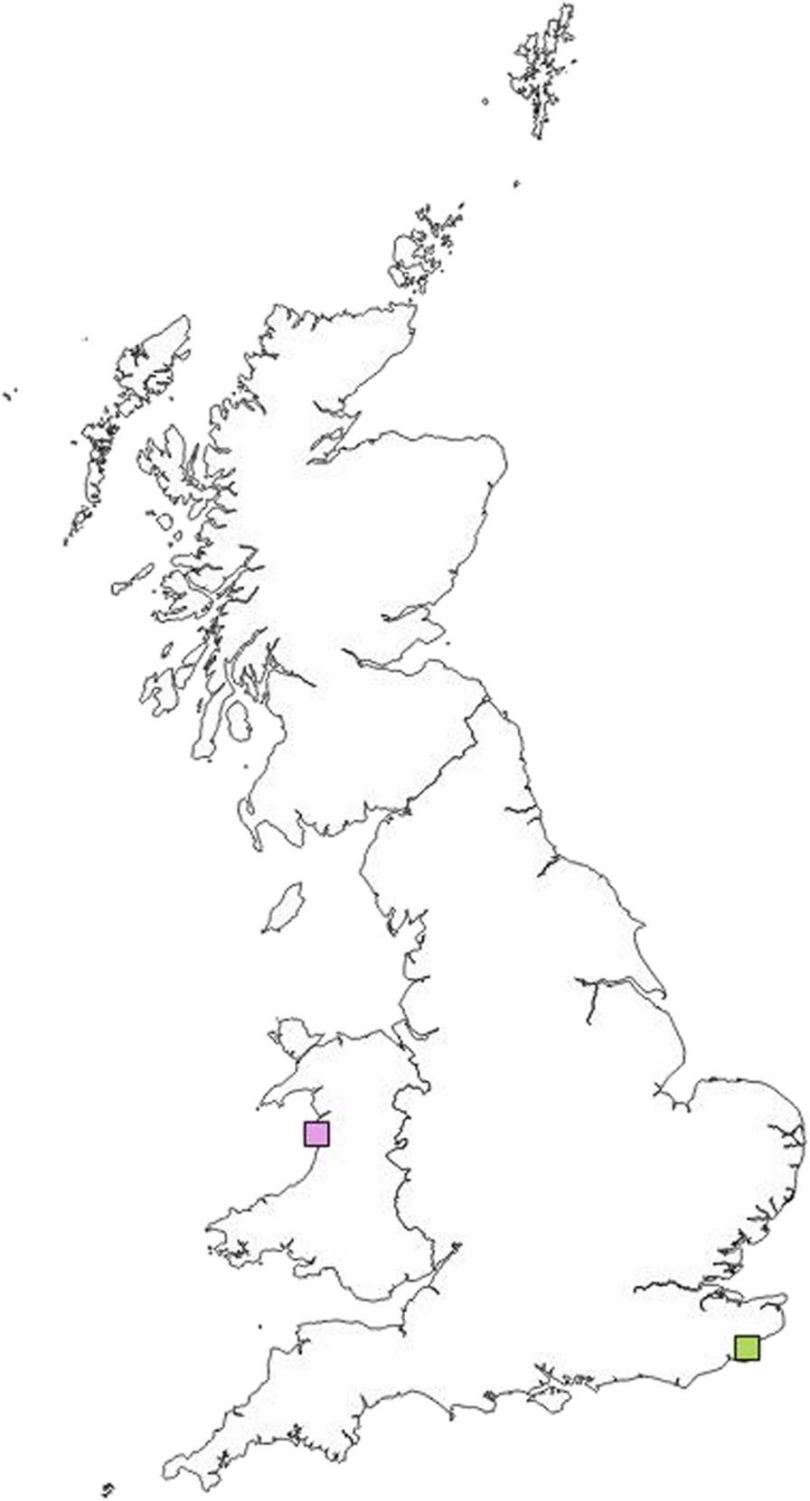
Location of ticks positive by cPCR for *Hepatozoon* species DNA. *Key* Green square, *H. felis*; pink square, *H. silvestris*

Two of the 540 ticks (0.4%) were positive by conventional PCR for *Hepatozoon* spp*.* DNA. The *Hepatozoon* spp. positive ticks were collected in Wales and the south-east of England (Fig. 5). DNA sequencing and BLAST analysis identified one *I. hexagonus* (0.2%) positive for *H. felis* and one *I. ricinus* (0.2%) positive for *H. silvestris* (Table 3). DNA sequencing revealed 90 and 100% identity, to sequences available in GenBank (accession numbers KY215817 and KX757032, respectively).

## Discussion

Relatively few studies have been performed describing the prevalence of tick-borne pathogens in ticks collected directly from cats compared to dogs. Here, the DNA samples from ticks collected from cats in the UK were analysed. As previously described, these ticks were mainly found in England and were screened for *Babesia* spp. and *Borrelia burgdorferi* (*sensu lato*) by PCR [3], with positive results for *Babesia* spp. found in 1.1% [*n* = 6 of 540 ticks, four “Babesia vulpes”, also known as *Babesia microti*-like, and two *Babesia venatorum*] and for *B. burgdorferi* (*s.l.*) in 1.9% (*n* = 10 of 540 ticks, six *Borrelia garinii* and four *Borrelia afzelii*), of the ticks. However, none of the ticks that were positive for *Babesia* spp. or *B. burgdorferi* (*s*.*l*.) in that study were positive by PCR for the selected pathogens described in the present study.

In a study performed in southern Italy in which 73 ticks from 15 cats were analysed using qPCR, the prevalence of *Bartonella* spp. in the ticks was 2.7% (qPCR for detection of *Bartonella* spp. targeting the internal transcribed spacer 1 (ITS1)), but no haemoplasma, *Ehrlichia/Anaplasma *spp. or *H. felis *DNA was detected [12]. In another study performed in south-west Italy, 132 ticks collected from 308 cats were analysed using qPCR; the prevalence of *B. clarridgeiae* in the ticks was 1.5% (qPCR for detection of *Bartonella* spp. targeting ITS1 followed by DNA sequencing) and the prevalence of *Ehrlichia canis *was 0.75% (qPCR for detection of *Ehrlichia/Anaplasma *spp. targeting *16S* rRNA followed by DNA sequencing), but no haemoplasma or *H. felis *DNA was detected [13]. In a study performed in Switzerland in which 71 ticks collected from 39 cats were screened for haemoplasma species using qPCR, the prevalence of “*Ca. *M. haemominutum” was 2.8% and no *M. haemofelis *and “*Ca*. M. turicensis” DNA was detected (qPCR for detection of haemoplasma species targeting *16S* rDNA) [14]. Other recent studies in which ticks were collected from both cats and dogs and screened for pathogens by PCR revealed a prevalence of *A. phagocytophilum *of 24.2% in Belgium (2373 ticks from 506 cats and 647 dogs) and 14.4% in Poland (93 ticks from 171 cats); however neither study indicated the prevalence of pathogens in only those ticks collected from cats [15, 16].

The pathogen prevalences found in our study can only be compared accurately to the two previous Italian studies and the Swiss study [12–14] as these results were obtained from ticks collected from domestic cats only (rather than combined dogs and cats as in the Belgium and Poland studies [15, 16]). *Anaplasma phagocytophilum* (0.9%) and *H. felis* (0.2%) were both detected in ticks in our study whilst none were found in the Italian studies [12, 13]. However, *Bartonella* spp. (1.3%) and *B. clarridgeiae* (0.2%) were less frequently detected in our study compared to the Italian studies (2.7 [12] and 1.5% [13], respectively). Additionally, “*Ca.* M. haemominutum” (0.6%) was less frequently identified in this study compared to the Swiss study (2.8%); however, we also detected M*. haemofelis* (0.2%) and “*Ca.* M. turicensis” (0.2%) DNA, which were not found in the Swiss study [14]. Possible explanations for these discrepancies include: differences in the tick populations analysed (i.e. ticks collected from cats in Italy and Switzerland were mostly *Rhipicephalus* spp., including *Rhipicephalus sanguineus* (*s*.*l.*), *Rhipicephalus pusillus* and *Rhipicephalus turanicus* and *Ixodes* spp. (including *Ixodes ventalloi* as well as *I. ricinus*))*,* different methods for DNA extraction and PCR assays and the very low pathogen prevalence detected in our study.

Studies investigating the prevalence of tick-borne pathogens in ticks collected from wild cats have been performed in Japan and Algeria [17–20]. DNA from *Anaplasma* spp., *Bartonella* spp., haemoplasma species and *Hepatozoon* spp. was identified in ticks from Japanese wild cats [17–19], whereas *Bartonella* spp. DNA was not identified in the ticks collected from Algerian wild cats, nor were these ticks tested for *Anaplasma* spp., haemoplasma species and *Hepatozoon* spp. DNA [20]. Thus, it is apparent that tick-borne pathogens may be of importance in wild cats too, although only limited data are available and comparisons with our data are difficult to make.

The rodent tick, *I. trianguliceps*, comprised 1.5% of the ticks collected from cats in the UK [3]. Two haemoplasmas, “*Ca.* M. haemominutum” and *M. haemofelis,* were identified in two separate *I. trianguliceps* samples. No other pathogens were detected in this tick species in our study and *Babesia* spp. and *Borrelia* spp. DNA were not detected in these ticks in an earlier study [3]. Overall, the prevalence of haemoplasma DNA in *I. trianguliceps* was 25% (2/8), although the number of ticks included is small. The three remaining samples positive for haemoplasma DNA were derived from *I. ricinus* ticks; resulting in a haemoplasma prevalence of 1.0% (3/308) in *I. ricinus*. These results suggest a possible association between feline haemoplasmas and *I. trianguliceps*, which warrants further investigation with larger numbers of ticks. The Swiss study identified two “*Ca.* M. haemominutum” in *Ixodes* spp.; however, the tick species were not determined. To the authors’ knowledge, this is the first report of haemoplasma detection in *I. trianguliceps* ticks.

In our study, the prevalence of *Hepatozoon* spp. was 0.4%. Sequencing confirmed the presence of one each of *H. felis* (*n* = 1 of 540 ticks, 0.2%) and *H. silvestris* (*n* = 1 of 540 ticks, 0.2%). Recently, *H. silvestris* was identified for the first time in European wild cats [21]. Meronts of *H. silvestris* were identified in cardiac and skeletal muscles associated with mild myocarditis and increased creatinine kinase activity, and pathogen DNA was also detected in the lungs and spleen of infected animals [21]. Interestingly, *H. felis* meronts were previously reported in cardiac and skeletal muscles in domestic cats without evidence of reactive inflammation suggestive of subclinical infection [22]. However, this is substantially different from *Hepatozoon americanum* infection reported in dogs and wildlife in the USA causing severe and painful pyogranulomatous myositis [22]. The present study is the first report of detection of *H. silvestris* in ticks collected from domestic cats. Additional studies are necessary to investigate further the pathogenicity and tropism of *H. silvestris* as compared to *H. felis* and *H. americanum*.

Detection of more than one pathogen in an individual tick DNA sample was not reported in our study. However, co-detection of tick-borne pathogens in *Ixodes* spp. ticks have been previously described; these have included *Babesia venatorum* and *Borrelia afzelii* in the UK [3], *A. phagocytophilum* with *Rickettsia helvetica* or *Borrelia afzelii* in Belgium [15], and dual, triple or quadruple infections with combinations of *Rickettsia* spp., *Babesia* spp., “*Candidatus* Neoehrlichia mikurensis” and *A. phagocytophilum* in Poland [16].

In our study, 88% of the tick samples were PCR positive for feline DNA. This result confirmed that most ticks were collected from cats after a blood meal. However, 71.7% of ticks that were classified as being unfed were PCR positive for feline DNA; it is likely that these ticks were removed from cats soon after they had started feeding and before they had engorged. Intriguingly, 16.4% of fully fed ticks and 8.6% of partially fed ticks were PCR negative for feline DNA (Table 2). Errors in DNA purification or the presence of PCR inhibitors were excluded by the positive IAC PCR results obtained on the tick DNA samples previously [3].

Our study had some limitations. Only cats that were presented to veterinarians were used as the source of ticks and so this cannot be considered a random sample of domestic cat ticks, although the cats that were examined were not brought into the veterinary surgery specifically for tick infestation. It could be argued that the cats sampled might also have been likely to receive preventative tick treatment, since they were under veterinary care. No IAC PCR for the presence of tick DNA was available, but an artificial IAC was included to confirm the presence of amplifiable DNA [3]. We were not able to use PCRs for the detection of feline *Anaplasma* or *Ehrlichia* spp. infection at a genus level on the tick DNA samples, due to the known presence of endogenous tick *Ehrlichia*/*Anaplasma* spp., as previously described [23], which confounds any positive results. Ideally, we would have simultaneously collected blood samples from the infested cats to determine whether these were the origin of the positive results in the collected ticks, but such samples were not available. Controlled studies are also required to determine whether ticks are true vectors for these pathogens.

## Conclusion

The results from this study provide important information on the prevalence of selected tick-borne pathogens in ticks found on cats, a research area infrequently studied. *Mycoplasma haemofelis,* “*Ca.* M. turicensis”, *A. phagocytophilum* and *H. felis* were found more commonly, and *Bartonella* spp., *B. clarridgeiae* and “*Ca.* M. haemominutum” less commonly, to similar studies on ticks from cats in Italy and Switzerland. This study also documents the first report of *H. silvestris* in ticks collected from domestic cats.

## Abbreviations

BLAST: Basic Local Alignment Search Tool
IAC: Internal amplification control
PCR: Polymerase chain reaction
qPCR: Quantitative polymerase chain reaction
